# Supporting intersecting cultural needs of gender and age by increasing cultural safety and humility for Housing First initiatives

**DOI:** 10.1186/s12889-023-15955-7

**Published:** 2023-05-30

**Authors:** Mei Lan Fang, Sarah L. Canham, Lupin Battersby

**Affiliations:** 1 School of Health Sciences, University of Dundee, City Campus, 11 Airlie Pl, Dundee, DD1 4HJ UK; 2 Department of Gerontology, Simon Fraser University, Burnaby, Canada; 3 College of Social Work, College of Architecture and Planning, University of Utah, Salt Lake City, USA; 4 Knowledge Mobilization Hub, Simon Fraser University, Burnaby, Canada

**Keywords:** Housing first, Cultural safety and humility, Participatory research, Homelessness, Service mapping

## Abstract

**Background:**

To sufficiently house and support persons experiencing homelessness (PEH), deeper understandings of the cultural appropriateness and responsiveness of community resources and the service delivery system is essential. In the case of Metro Vancouver, Canada, the cultural appropriateness and responsiveness of Housing First as a service model for supporting PEH was explored.

**Methods:**

Local service providers and stakeholders (n = 52) participated in three full day service-mapping workshops to identify Housing First supports for older adults, youth, and women experiencing homelessness, as part of a municipal-wide participatory and action-oriented study. Data were analyzed using a structured framework thematic analysis approach and cultural safety and humility lenses.

**Results:**

We generated three key themes: (i) insufficient built environments create challenges across gender and age, (ii) cultural safety and humility concerns at the intersection of gender and age, and (iii) implications for a culturally-responsive Housing First implementation.

**Conclusions:**

Findings informed the development of a Culturally-Responsive Planning resource to support housing, health, and social service providers who are implementing Housing First initiatives.

**Supplementary Information:**

The online version contains supplementary material available at 10.1186/s12889-023-15955-7.

## Introduction

The COVID-19 pandemic brought to light longstanding public health and community planning questions on how best to house and serve the diverse population of people experiencing homelessness (PEH) [1]. On any given night, there are more than 580,000 Americans [2] and over 35,000 Canadians [3] experiencing homelessness. Homelessness can be seen in every major metropolitan city in the United States and Canada [1], including Metro Vancouver (also known as Greater Vancouver), a region with among the highest rates of homelessness in Canada [3]. Moreover, the face of homelessness is changing as certain sub-populations are increasingly likely to experience homelessness, including older adults – defined as persons aged 50 + due to accelerated aging [4, 5] – and youth [6]. Since Metro Vancouver’s housing market surge began in 2016, housing insecurity has disproportionately impacted youth, older adults, and women with limited financial resources [7].

Given the strong evidence that Housing First approaches improve health and housing outcomes for persons with severe mental illness who have experienced chronic homelessness [8, 9], federal investments in homelessness prevention and management have prioritized Housing First initiatives [10, 11]. Housing First advocates for the use of a systems-approach to homelessness with providers working in a collective, multi-system, and cross-sectoral manner [12, 13]. The six Housing First principles identified by the Government of Canada [14] include: (1) provision of rapid housing with supports; (2) offering clients’ choice in housing; (3) separating housing from other services; (4) providing clients with tenancy rights and responsibilities; (5) integrating housing into the community; and (6) strengthening and building on the skills and abilities of the client, based on self-determined goals. As opposed to housing programs that require clients to receive ‘treatment first,’ Housing First prioritizes stable housing prior to working toward other self-sufficiency goals [15, 16]. Moreover, sufficient access to housing and supports is essential for the successful delivery of Housing First, while insufficient resources is a key barrier to implementation [9].

To adapt and enhance the Housing First approach in Canada, a large-scale research study, namely the At Home/Chez Soi Demonstration Project was conducted in Canada between 2009 and 2013 to investigate the effectiveness of the Housing First approach for people experiencing homelessness and mental health issues [17]. The project was implemented in five cities: Vancouver, Winnipeg, Toronto, Montreal, and Moncton. A key aspect of the project was its attempt to modify the Housing First approach to different populations, including Indigenous communities, racialized communities, and rural communities [17].

For Indigenous communities, recognizing the overrepresentation of Indigenous people among the homeless population and the importance of culturally appropriate interventions, the At Home/Chez Soi project incorporated culturally-specific programming for Indigenous participants [17]. For racialized communities, the project acknowledged the unique challenges faced by racialized individuals, including systemic racism, discrimination, and cultural barriers. In response, the At Home/Chez Soi project emphasized the importance of cultural competency among staff members and tailored its services to better address the needs of racialized participants [17]. Last, regarding rural communities, the At Home/Chez Soi project included Moncton, a smaller city with a mix of urban and rural populations [17]. Adapting the Housing First approach to rural communities presented unique challenges, such as limited availability of affordable housing, transportation barriers, and fewer support services. To address these challenges, the project focused on enhancing collaboration between service providers, increasing the flexibility of support services, and providing transportation assistance when needed [17].

In Metro Vancouver, the division of responsibilities across 21 municipalities, one electoral area, and one Treaty First Nation, two health authorities, and five municipal forces leads to different policies and practices, which challenges the systems-approach to Housing First.

In Metro Vancouver, Housing First funds are used by service agencies to support persons who have experienced chronic and/or episodic homelessness—that is, staying 180 + nights in a shelter or place unfit for human habitation or having had 3 + episodes of homelessness in the past year [18]. Despite the significant body of literature on Housing First for individuals experiencing chronic or episodic homelessness [3, 19], there is limited information on the responsiveness of Housing First programs for sub-populations of PEH with distinct needs, including older adults and newly homeless individuals [10, 20]. Nevertheless, it is imperative that housing, homelessness, and service delivery systems address individual barriers and meet diverse needs by considering a person’s combined social and cultural identities and positions [21]. Thus, there is a need for culturally-responsive systems within which culturally-appropriate community resources function [22].

Being culturally-appropriate and -responsive refers to: (1) understanding and tailoring to the needs of an individual’s culture, including values, beliefs, meanings, and expressions shaped by varied sociocultural environments; and (2) responding to diverse cultural needs by honoring and accounting for cultural and linguistic differences to earn and maintain trust with active endeavors to address biases, assumptions, stereotypes, and prejudices for the development and delivery of quality services and supports [23, 24]. For instance, in nursing, consistent efforts to review, critically examine, and reflect on what it means to be culturally-appropriate and -responsive has been paramount to providing safe, quality care to diverse patients [24].

Notions of cultural safety and humility stem from the umbrella of cultural competence [25], while seeking to rectify the overvaluation of Eurocentric knowledge, beliefs, and practices by moving beyond a simple demonstration of competence or knowledge about ‘the other’ [26] towards enabling equality of opportunity and human interactions. Here, cultural humility refers to enhanced ways of working by housing, health, or social service professionals to ensure safety for PEH. Cultural safety emerged from the nursing field [27] as a concept aimed at improving the safety of minoritized individuals within the context of healthcare and social service provision [28–30]. Alongside notions of cultural safety, cultural humility emphasizes the power held by providers in relation to clients and the need for providers to prioritize humility, have cultural knowledge about their clients, and to understand and empathize with clients’ beliefs, understandings, and experiences of their social and health realities [31].

Informed by tenets of cultural safety and cultural humility – conceptual models to enable culturally-appropriate and -responsive health and social care programming and delivery [27, 32] – we examined service providers’ perspectives on the cultural appropriateness and responsiveness of the Housing First approach for supporting several sub-populations of PEH. Using Metro Vancouver as a case study, we examined: How can tenants of cultural safety and cultural humility be applied to support sub-populations of youth, older adults, and women who are experiencing homelessness? From the findings, we developed a *Culturally-Responsive Planning* resource, suitable for use across different geographical contexts, to assist in culturally-responsive planning for housing, health, and social service providers when implementing Housing First initiatives.

### Research context

In Metro Vancouver, the division of responsibilities across 21 municipalities, one electoral area, and one Treaty First Nation, two health authorities, and five municipal forces leads to different policies and practices, which challenges the systems-approach to Housing First. In Metro Vancouver, Housing First funds are used by service agencies to support persons who have experienced chronic and/or episodic homelessness—that is, staying 180 + nights in a shelter or place unfit for human habitation or having had 3 + episodes of homelessness in the past year [18].

Prior to Canham and colleagues’ [33] study, there was no formal documentation of Housing First resource differences between and within Metro Vancouver communities, which created difficulties for service sectors when advocating for resources to support PEH. In order to inform recommendations to improve Housing First service delivery in Metro Vancouver, a community-based participatory research (CBPR) study was undertaken to understand Metro Vancouver’s homelessness-related support system. Through preliminary data analysis from this parent study, the authors identified that housing, health, and social service challenges of youth, older adults, and women were shaped by intersecting cultural needs of gender and age. To optimize Housing First for these sub-populations, planning for housing, health, and social services must be culturally-responsive to the unique needs of PEH in early life, later life, and in the gendered role of women. To identify the responsiveness of Housing First to support the diverse sub-cultures of youth, older adults, and women who are experiencing homelessness, we used the lenses of cultural safety and cultural humility to identify housing, health, and social support needs, as well as variations in power differentials between providers and clients. We contextualize findings according to understanding Housing First as a set of guiding principles for housing and supports as opposed to its operationalization in practice (i.e., design and administration of programs and policies).

## Methods

### Study design

Analysis of a subset of a larger dataset that sought to understand the housing-related support system in ten communities and with three sub-populations across Metro Vancouver was conducted. Guided by CBPR principles and methods, the parent study was conducted in collaboration with the Greater Vancouver Shelter Strategy (GVSS) and bc211 (a local information and referral service agency) using a community mapping method [33, 34], inspired by Participatory Rural Appraisal [35]. The focus on the sub-populations of of youth, older adults, and women was driven by community partners and funders who had highlighted clear gaps in knowledge of the contextual challenges of homelessness experienced by these groups. Ethics approval was provided by Simon Fraser University’s Institutional Review Board and participant names were not linked to data collected to protect their identity.

**Fig. 1:**
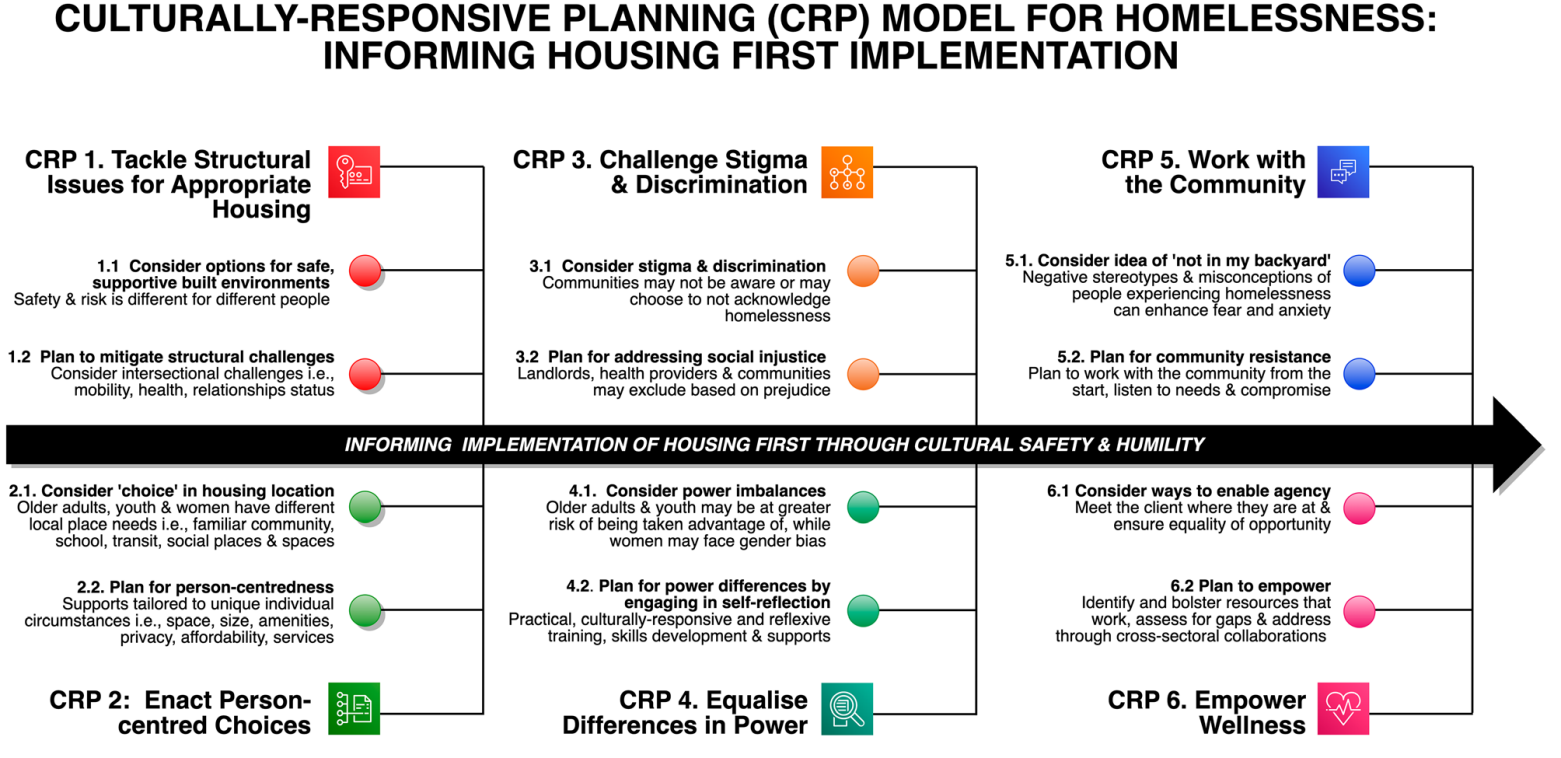
Visual model depicting six key culturally-responsive principles to inform implementation of Housing First. Developed based on a Metro Vancouver case study of homelessness using cultural safety and humility as an analytical framework. Application of this model is supported by the Culturally-Responsive Planning Tool (Table 1)

In the parent study, community mapping workshops produced rich data on available Housing First services and supports, specifically for PEH, and the ways in which these functioned in the housing system (i.e., systemic barriers and facilitators for change). Informed by relevant elements from the full dataset [34], the current study examined data from the three population-specific workshops to understand the housing and social service challenges, needs, and nuances of youth, older adults, and women. Here, women includes persons who hold this gender identity. However, as indicated in our findings, not all women-serving services use such an inclusive definition to meet the needs of all women.

In population-specific workshops that focused on sub-populations (youth, older adults, and women), participants from various municipalities across Metro Vancouver convened to discuss the issues unique to seniors, women, or youth experiencing homelessness. To facilitate this, one geographic map from each of the 10 Greater Vancouver municipal regions was displayed on the walls around the room. Throughout the workshops, participants engaged in the mapping process by placing sticky notes on the pre-printed maps, which visually depicted the locations of housing support services and resources in their respective communities.

Researchers facilitated discussions centered on the functionality of the mapped services across different regions, addressing aspects such as accessibility, gaps, communication, and more. Example questions explored the difficulties in using these services and supports, factors that facilitate access to community services and supports, gaps in service provision, and alternative sources for resources unavailable in the area. To enhance the quality of the collected data, researchers documented key observations from each workshop through field notes and post-event reflective summaries.

### Participants and recruitment

Participants were recruited by email invitations sent to members of private mailing lists organized by the Greater Vancouver Regional Steering Committee on Homelessness and GVSS, and via a database maintained by bc211. Invitations encouraged providers to invite a client from their organization to join them. Individuals were eligible to participate if they were 19 years or older, able to provide informed consent, and either a current or potential Housing First provider in Metro Vancouver or a youth, older adult, or woman client of a Housing First provider. Fifty-two individuals, 7 of whom identified as having lived or living experience of homelessness, participated in one of three population-specific mapping workshops in Metro Vancouver [33]: 16 at the youth-specific workshop, 17 at the older adult-specific workshop, and 19 at the women-specific workshop. The majority of workshop participants were providers representing government agencies, housing associations, community centers, charitable organizations, and health authorities. While we sought to engage more clients, we were not successful and recognize this as a limitation of the study. However, the tacit experience of service providers is valuable given their significant experience supporting clients, navigating challenging systems, and in some cases through their own lived experience of housing instability and homelessness. Indeed, the precarious line between housed and homeless is not uncommon in Metro Vancouver [36].

### Data collection

**Fig. 2:**
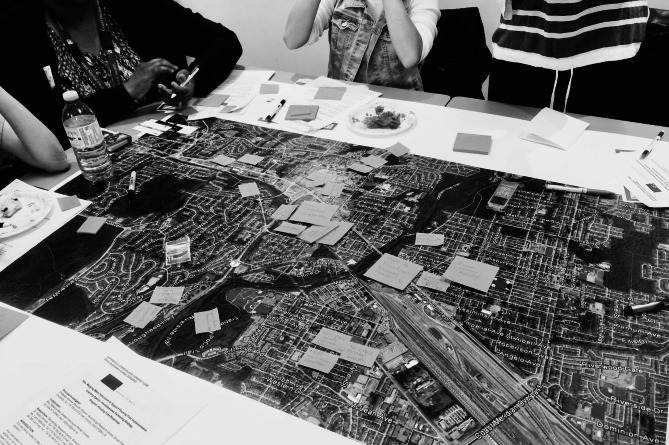
In-depth small group community mapping exercise

**Fig. 3:**
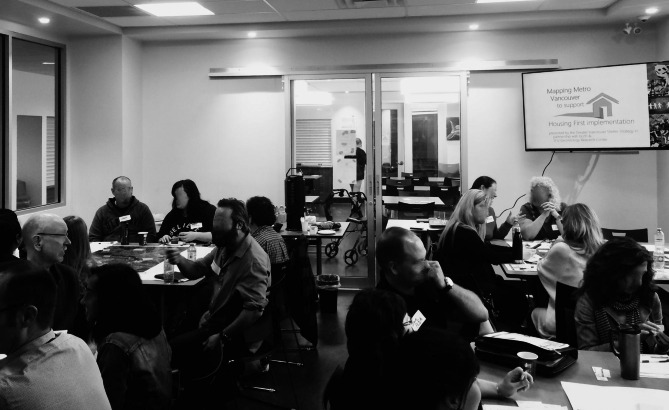
Large group roundtable discussions

Workshops began with a mapping exercise, where participants applied notes to pre-printed maps indicating where housing, health, and social resources were located in their communities. This activity was followed by a group case study analysis and in-depth small group and large group roundtable discussions (Figs. 2 and 3).

**Table 1:** Culturally-Responsive Planning (CRP) Tool

Guiding Principles	Recommendations for Planning	Considerations, Questions and Resources
*Implement HF 1: Rapid housing with supports* *Supported by* *CRP 1: Tackle structural issues for appropriate housing*	*1.1 Consider options for safe, supportive built environments* • Consult with local housing, health, and social service providers to identify needs of different populations, and local issues and benefits (risks, resistance, resources, transportation, schools, housing options) • Work with providers to develop culturally-responsive, population-specific housing and programming based on needs for gender-safe spaces, safe living (perhaps in group settings) for youth, senior-safe spaces, substance dependence services, youth awareness and mentorship, life-planning for youths, population-specific skills development, and mental health support such as counseling for past trauma *1.2 Plan to mitigate structural challenges* • Work with government agencies to identify population-specific subsidy needs; ensure easy and supportive application processes are in place; collaborate with housing providers to review criteria for housing for specific cases	*Cultural safety and humility consideration* • Recognize how the system is built on a design informed by a dominant culture, gender, or age group that is situated in positions of power, thus tailoring to the needs of individuals with more access to housing and related supports and services *Reflexive question(s) for responsive planning* • What planning tasks, relationships, or situations have been neglected in everyday practice at work and in the community when addressing needs of persons experiencing homelessness? • What other ways can structural challenges to housing be viewed (i.e., for youth, older adults, and women)? *Suggested existing resource* • Canadian Housing First Toolkit [9]
*Implement HF 2: Offering clients’ choice in housing* *Supported by* *CRP 2: Enact person-centered* *choices*	*2.1 Consider for ‘choice’* • Explore with government agencies and developers: mixed-use housing and needs of community; available housing resources assessing whether needs are met; sustainability of housing and resources; and intended & unintended impact of new housing developments (i.e., neighborhood gentrification) *2.2 Plan for person-centeredness* • Resource cultural and other knowledge brokers with communication capability (ideally across cultures) and understandings of different cultural norms, values, and beliefs	*Reflexive cultural safety and humility consideration* • Reflect on how your own position might limit understandings of the community and the needs of individuals *Question(s) for responsive planning* • What can planners do to be more aware of their positions and associated values, beliefs, and practices? • How do planners consider the diverse cultural and individual needs of clients so that their practice is person-centered? *Suggested existing resource* • Housing First Europe Guide [40]
*Implement HF 3: Separating housing provision from other services* *Supported by CRP3: Challenge stigma and discrimination*	*3.1 Consider for stigma and discrimination* • Consider how some communities may not be aware of or refuse to acknowledge issues of homelessness; and some persons experiencing homelessness (e.g., youth, older adults and women) may be at greater risk of stigma from landlords and healthcare providers, making the journey to stability more difficult *3.2 Plan for addressing social injustice* • Explore ways to tackle clients’ experiences of stigma and discrimination to first point of access; develop services and resources that all people can benefit from (e.g., walkability, accessibility, local amenities); normalize diverse social experiences (e.g., substance use, single motherhood, being poor, living with co-morbidities); and develop and deliver cultural sensitivity training involving introspective reflexive-working	*Cultural safety and humility consideration* • Create culturally-responsive housing and supports that considers the culturally diverse needs of youth, older adults, and women *Reflexive questions for responsive planning* • What culturally-responsive housing, health, and social services are available to support diverse sub-populations of persons experiencing homelessness? • How can these be shaped to become culturally-responsive? *Suggested existing resource* • Planning for Equity Policy Guide [41]
*Implement HF 4: Providing tenancy* *rights & responsibilities* *Supported by* *CRP 4: Equalize differences in power*	*4.1 Consider power imbalances* • Consider that some persons experiencing homelessness may have less ability to enact agency and power when accessing housing and support services (such as single mothers, women fleeing violence, youth, older adults, persons identifying as Aboriginal, transgender men and women, and persons living with co-morbidities, including mental health and substance use disorders) *4.2 Plan for power differences by engaging in self-reflection* Work across sectors to explore more options for empathic and respectful housing providers / landlords; mechanisms to create supportive living environments with accessible and tailored supports; and develop cross-sector partnerships to enable multisystem supports for clients with complex needs	*Cultural safety and humility consideration* • Reflect on how power imbalances between planners, service providers, and clients can escalate when there are differences in age, gender, education, income, language, culture, and ability that contribute to diverging socio-cultural beliefs *Reflexive questions for culturally-responsive planning* • What culturally-responsive training and supports do planners and service providers need to be reflective and aware of their positions of power? How can planners and service providers be better supported? *Suggested existing resource* Reflexive Strategies in Planning [42]
*Implement HF 5: Integrating housing into the community* *Supported by* *CRP 5: Work with the community*	*5.1 Consider idea of ‘not in my backyard’* • Consider how some landlords may avoid renting to youth, single mothers, older adults, visible minorities, people on social assistance, people with mental health or substance use disorders, or persons with physical disabilities *5.2 Plan for community resistance* Develop mechanisms to: (1) work with communities on new housing projects (i.e., consultation, transparency) towards buy in; (2) partner with housing, health, and social service providers across sectors and communities to create integrated working and shared resourcing; (3) collaborate with local council / government to leverage more funding and incentives for people who live and work in the community; and (4) deliver knowledge and education on person-centeredness for people who live and work in the community	*Cultural safety and humility consideration* • Address negative stereotypes and misconceptions of individuals and groups fueled by historical and political contexts that have influenced and normalized structural inequity *Reflexive questions for responsive planning* • How can planning include strategies for encouraging community awareness of historical and political contexts that have shaped homelessness and lived experiences? • How can planning mechanisms promote responsibility and care for all members of the community? *Suggested existing resource* Model for Culturally-Responsive Housing Provision [43]
*Implement HF 6: Strength-based & promoting self-sufficiency* *Supported by* *CRP 6: Empower Wellness*	*6.1 Consider ways to enable agency* • Consider strategies for resourcing ambassadors or navigators and wrap-around services that support and empower clients to ensure success; and onsite and person-centered services and staff to increase clients’ sense of accessibility, safety, and belongingness *6.2 Plan to empower* Develop a community empowerment plan focused on working with housing, government and health sectors, clients, and family members towards solutions for ambassadors or navigators and wrap-around services; onsite supports; transportation accessibility; safe access to variety of services. The plan should leverage existing resources, while planning for the lack of other resources and identify ways to build skills, confidence, emotional and psychological resilience and maintain self-care	*Cultural safety and humility consideration* • Build supportive environments to empower clients by focusing and building on clients’ strengths; respecting and learning clients’ values, beliefs, and everyday realities; and working both with and for them *Reflexive questions for responsive planning* • What is your knowledge about the community and people you are working to help? What are ways to work with and for them, building on their strengths? • Are there existing resources available to develop and implement a community empowerment plan? What is already available and what more is needed? *Suggested existing resource* Developing a Neighborhood Empowerment Plan [44]

Two researchers were at each table for notetaking and facilitation. The mapping process resulted in 18 h of rich audio data and visual representations of geographical locations where resources were sufficient for distinct groups or where there were gaps. During the mapping activity, a member of the research team facilitated discussions on how services functioned in different regions (i.e., accessibility, availability, and communication). Example prompt questions included: *What difficulties are there in using these services and supports? What helps you access resources in the community? Where are there gaps in service provision? How do you get resources that are unavailable in your area?* To enhance depth of knowledge and inform data interpretation, alongside documenting methodological strengths and limitations, researchers kept field notes and produced reflexive summaries during and after the workshops. Workshop discussions were digitally-recorded (with informed consent) and transcribed.

### Data analysis

Data were thematically analyzed using a structured framework approach [37], in NVivo 12. Two researchers were involved in all stages of data analysis [38]. In Phase 1, a coding framework (Supplementary File 1) was systematically developed according to tenets of cultural safety and humility [32, 39]. The goal was to develop themes independent of preconceived notions held by members of the research team and to enable extraction of relevant information informed by the cultural lenses. Using the structured framework, Phase 2 of analysis involved reading and re-reading transcripts for the purpose of data familiarization. In Phase 3, transcripts were analyzed using the framework by case and by code [37]. Phase 4 involved arranging, analyzing, and organizing themes and incorporating feedback from group analysis meetings. In Phase 5, a second researcher conducted an independent review of the coding to further refine the themes. Phase 6 involved all three authors discussing the final set of themes to reach consensus.

## Findings

We identified three overarching themes: (1) Environmental challenges across diverse gender and age groups, (2) Cultural safety and humility considerations at the intersection of gender and age, and housing insecurity, and (3) Supporting culturally-responsive Housing First implementation. Details of thematic concepts and supporting data from the data analysis can be found in Supplementary Files 2–4, to further illuminate the overarching themes.

### Theme 1: environmental challenges across diverse gender and age groups

Participants highlighted the challenge of addressing population-specific needs in the context of a fundamental shortage of affordable and accessible housing suitable for diverse populations. Although one of the core principles of Housing First is to provide people experiencing homelessness (PEH) with choices in housing, the limited options available constrain this choice significantly. As one participant from the women-specific workshop articulated:

There’s that overall lack of affordable housing that poses a barrier for any typical family to access. So, until we address the lack of housing, then we cannot look into the particular services for individuals with particular needs.

This finding underscores the need to align and integrate the elements and implications of the Housing First approach by ensuring that adequate affordable and accessible housing options are made available. Addressing this fundamental issue will enable more effective implementation of population-specific services and support the ultimate goal of Housing First – providing stable housing to PEH.

The challenge of providing population-specific resources to effectively support the health and well-being of PEH was also highlighted. During the women-specific workshop, attendees reported a lack of adequate women’s-only services and suggested that a more targeted ‘women’s-only’ model might better serve this population. One participant noted:

Insufficient women-only services, combined with the broad categorization of ‘women,‘ can overlook subgroups within that category (e.g., older women, immigrant and refugee women, Indigenous women, transgender women). Sometimes, women-only services aren’t specific enough […] more tailored services are needed.

Another participant reinforced this idea by stating, “given the prevalence of violence, it’s crucial to have women-specific resources.“ However, an additional challenge raised by a participant was the operational barriers of some women-specific services that exclude transgender women:

Recently, we have encountered more transgender individuals seeking help. However, it’s challenging to find support for transgender women or men because services are often divided between ‘men’ and ‘women.‘ I’ve even encountered cases where a service refused help because the person wasn’t living 24 h as a woman.

Regarding implications for the Housing First approach, it is essential to address the gaps in population-specific resources. By developing and implementing services that cater to the unique needs of different subgroups, including women, older adults, Indigenous people, immigrants, and the LGBTQ + community, Housing First can more effectively promote stable housing and improved well-being for all PEH.

Participants also discussed the drawbacks of using a ‘one-size fits all’ approach when designing environments for diverse groups of people experiencing homelessness (PEH) with distinct social identities and circumstances. The importance of safe and secure environments for sub-populations such as youth, older adults, and women, noting that some PEH may choose to remain unsheltered due to fear, anxiety, and mobility challenges experienced in homeless shelters was emphasized. One participant from the women-specific workshop shared an example of the compounded experience of being a single older woman:

Many of them [PEH] have said they would prefer to live and sleep on the streets rather than go to a shelter […] because they perceive some shelters to be very dangerous. I know of single senior women who went to a shelter and felt unsafe.“

As such, it is crucial to consider the unique needs and preferences of different sub-populations of PEH when designing environments and services when as it relates to Housing First. This could involve creating specialized shelters or housing programs tailored to the safety and well-being of specific groups, such as youth, older adults, or women.

A final environmental challenge identified by participants was transportation, which was emphasized as a critical environmental factor that could either facilitate or hinder access to housing and services, depending on costs and the connectivity of transit networks, particularly as it relates to enhancing Housing First. Ensuring that affordable, safe and accessible transit options are available to PEH will facilitate better access to the necessary resources and support. As one participant from the women-specific workshop explained:

When you look at it – the Downtown East Side – all those resources; then you look at Greater Vancouver, there are not many options […]. People are going on a regular basis, every day, to those resources, rather than travel to many different places where they don’t even have any transportation.

In addition to transit networks, participants highlighted the need for an integrated and centralized system of services that caters to the diverse needs of people experiencing homelessness (PEH). For instance, placing services in areas that are safe, secure, and away from potential triggers (e.g., liquor stores or areas with high crime rates) can help promote recovery and well-being for individuals accessing these services. A participant from the women-specific workshop expressed the following:

We need a one-stop shop instead of spread-out resources. While it is valuable for other communities to share the load, having services dispersed is challenging. Clustering services makes sense, but their locations should be carefully chosen. For example, don’t place a recovery house next to a liquor store or a transition house near alleyways where violence occurs. Temptations are everywhere, yet individuals are expected to recover successfully.“

Housing First programs should work towards developing a more integrated and centralized system of services to cater to the diverse needs of PEH. By creating a one-stop shop or resource hubs that bring together various services in a single location, individuals can more efficiently access the support they need. This approach not only streamlines the process for PEH but also fosters better coordination among service providers.

### Theme 2: Cultural safety and humility considerations at the intersection of gender, age, and housing insecurity

Participants identified various pathways to homelessness, unique challenges faced by different demographics, experiences of multiple layers of social inequity, client-provider power imbalances, and discrimination and stigmatization. The issues discussed include challenges for younger women aging out of the foster care system, the impact of culture on women’s decision to remain in abusive relationships, the need to empower youth, unique pathways to homelessness for older adults, and experiences of intersectional homelessness.

At the intersection of women and youth, participants identified challenges for younger women who have recently aged out of the foster care system. Lack of financial resources and work experience was reported to be a risk factor leading to exploitative sex work or becoming reliant on abusive partners for financial or housing security. As one participant from the youth-specific workshop stated:

Sometimes women at a young age will turn to prostitution because when they age out [of the foster care system], or when they turn 18 with a family in foster care, if they’re in low-income housing, they have to pay rent or otherwise they have to get out. I was in a situation where I lived with my mom. She was on assistance my whole life. When I turned 18, they said I needed to pay, or I need to move somewhere else.

I actually called escort places thinking this is my only option. I was only 18 years old. And this is a common thing. Our young girls become targets at a young age.

Also influential in some women’s decision to remain in abusive relationships and shape experiences of homelessness, a participant from the women-specific workshop reported:

Culture is another big barrier. I know that in Indian [South Asian] culture, it’s not normal to leave […] the abusive relationships. Even if it’s not necessarily abusive, it’s not a good or healthy relationship; it’s still really hard to leave.

Regarding the culture of youth who are experiencing homelessness, participants identified the need to empower youth to make their own decisions rather than strip youth of their agency, which can have detrimental outcomes. Empowering individuals experiencing homelessness, particularly youth, by involving them in decision-making processes and providing them with resources to develop autonomy and self-sufficiency was highlighted as crucial for enhancing Housing First programs. A youth-specific workshop participant stated:

From my experiences dealing with youth, it’s too much all at once and then they just shut down, so that’s why there might be some struggles for school. So, I make sure this youth is involved in their plan, because a lot of the times, up until 19, everything is made up for them—“you’re gonna do this, you’re gonna do that”—and then it’s kind of like the ball is dropped and they’re like, “I don’t know what to do here” […]. That’s the biggest thing, just hearing the youth, “What do you wanna do? I know you’ve been told what you should do, but what do you actually wanna do?”

Related, a common pathway to homelessness for youth is when they age out of foster care and need to find housing with limited resources or experience the combined stigma of being young and on public assistance.

Participants also described unique pathways to homelessness for women who may not meet the typical inclusion criteria for Housing First supports. For women, concerns about their safety and the safety of their children were identified as shaping their approach to accessing housing and resources. Mothers were reported to worry about children being removed from their care, being ineligible for program support (i.e., because they were not chronically homeless or children are not allowed, etc.), and having other safety concerns. A women-specific workshop participant stated:

There are other issues with their children, too. They could be at-risk of being taken. So, then they don’t want to reach out to these places [shelters] because what if their kids are taken because they’re homeless, because they’re putting their kids at risk? That happens a lot.

The pathway to homelessness for older adults was identified as vastly different from that of youth. Participants indicated that older adults are increasingly unable to remain in their homes due to heightened property taxes, while living on fixed incomes, and challenges maintaining physical upkeep. Challenges of aging, including illness or the death of a spouse, exacerbated these issues. Moreover, older adults who are newly homeless or have a short history of homelessness were identified as not meeting eligibility for Housing First services, which prioritizes adults who have experienced chronic homelessness. A senior-specific workshop participant stated:

Housing First doesn’t really benefit seniors. Persons would have to become homeless first to be able to benefit from this. That is, as I see it, a real flaw in the system. You want to be better able to support a person who is on fixed income, who has income but still cannot afford to live where they are, and know what we can do to help them stay at home, so that they don’t become somebody who is homeless and then have to access services; especially if you think about the cost of that versus the cost of somebody who remains in their home.

This finding emphasizes the importance of eviction prevention measures for older adults, as the current Housing First approach may not be as beneficial to seniors as it is to other demographics. The statement by the senior-specific workshop participant highlights the limitations of Housing First for older adults, as they often need support before becoming homeless.

The participant’s concern lies in the system’s focus on individuals who are already homeless, rather than addressing the needs of older adults on fixed incomes who struggle to afford their current living situation. Eviction prevention measures for older adults are crucial because they can help seniors maintain their current housing and avoid the detrimental effects of homelessness. In addition, preventing evictions is often more cost-effective than providing services to someone who has already become homeless.

As well, at the heart of this finding and the following sections, which discusses a common thread to homelessness for PEH shaped by various social intersections is the need for Housing First programs to prioritize prevention and early intervention strategies to address the root causes of homelessness and support at-risk individuals before they become homeless. This includes working with schools, community organizations, and other population specific service providers to identify and assist at-risk individuals across different demographics.

For instance, another pathway to homelessness for older adults described by participants was elder abuse and poor treatment by adult children if an elder becomes ill or is no longer needed to care for grandchildren. A senior-specific workshop participant reported how an older woman was abandoned at a hospital by her family:

Some family members dropped this older woman off [at the hospital], because they said they couldn’t take care of her anymore. But, she had no real health issues besides maybe some dementia […]. We used to see a lot of elder abuse, financial especially, taking their money and then just dropping them off.

Our analysis revealed that experiences of homelessness are intersectional. For example, participants reported shortened life expectancies for PEH who are Indigenous and/or older and/or struggle with substance use dependency. Similarly, for some older ethnocultural adults experiencing homelessness, the inability to communicate in English challenged access to services and put non-English speakers in vulnerable positions.

I have one senior who is male, he comes out of a shelter every morning—I think before ten o’clock—and he is wandering around. He doesn’t want to go back there […]. His stuff has been stolen and he was the only person who spoke Mandarin in that shelter, no one speaks languages other than English.

On further interpretation this excerpt, this service provider highlights the need for more collaboration with other service providers and community organizations to create a holistic and integrated support network for individuals at the intersection of gender, age, and other factors contributing to homelessness to enhance Housing First programs. This may involve providing targeted support for specific groups, such as women, youth, and older adults, to address their unique needs and challenges.

Another challenge for PEH is the power imbalance between homeless clients and providers who are often ill-equipped to provide culturally-safe and responsive care.

It’s frustrating dealing with hospitals. The nurses and the doctors need some kind of training on sensitivity […]. And, even sometimes the paramedics. There are some paramedics who are amazing […], but some paramedics who are really disrespectful to women, we’ve seen it. It’s terrible how the women get treated.

Discrimination and power imbalances can also be seen in the requirement of homeless clients to retell experiences of trauma, which can be exhausting and discouraging, to different providers to receive new services.

Within mental health services or child and youth mental health services, obviously, there’s a wait time–there’s a whole referral process and sometimes you have to retell your story of trauma or whatever over and over to your counsellor or to your GP [general practitioner] or to your psychiatrist. I think you just lose faith in the system and trust.

Power imbalances are further observed through the discrimination and stigmatization of intersectional social identities. For instance, youth can be stigmatized by landlords, as a youth-specific workshop participant indicated:

What I’ve noticed is there’s a lot of stigma of youth who have been in [foster] care. When I call in, I’ll be honest, I don’t say it’s for youth. I’ll be, like ‘Oh, you got a suite available, can I come and see it?’ and then I’ll go with the youth. And we actually provide a letter, saying they’re in our program and they are engaged in our program, and we do check-ins, we do program planning, things like that. So, it has helped, but I still feel that there is a lot of pull-back from landlords.

Landlords were also reported to discriminate against individuals on income assistance, single mothers, and individuals who identify as or appear ‘Asian’ or ‘Aboriginal’.

I saw a news release before, and it said the number one thing landlords don’t want to rent to is single moms. The next one is First Nations people. The next one was Asian people. And I think the other one was people on income assistance.

And I was like, no wonder I’m not getting any housing, I’m a single mom, I’m First Nations, I could pass for Asian, and I’m on income assistance. I had so much against me.

When interpreting the aforementioned excerpts by participants, the implication here is that Housing First programs should work to reduce power imbalances between clients and providers, fostering an environment that is free from discrimination and stigmatization. This can be achieved through training, open communication, and feedback channels between clients and service providers.

It is important to note that, homelessness is disproportionately experienced by Indigenous, black, and people of colour and can be particularly stigmatizing. Not only are individuals experiencing homelessness stigmatized by the public because of their homeless status, but they are also stigmatized for being a ‘visible minority’ by health and social care systems. Housing First programs should actively work towards combatting discrimination and stigmatization experienced by individuals from diverse backgrounds, both within the program and in interactions with external service providers. This can be achieved through advocacy, education, and creating safe spaces for clients to share their experiences.

### Theme 3: supporting culturally-responsive housing first implementation

The final theme highlights systems-level considerations to enhance Housing First with emphasis on being responsive to cultural nuances behind diverse experiences of homelessness, including upstream prevention and resources; prioritizing diversity in support staff; and enhanced partnering and tailoring of resources to accommodate diverse PEH.

Across the three workshops, participants emphasized the need for increased funding to maintain and increase population-specific resources for housing, health, and social services. A participant from women-specific workshop stated:

Increased funding for housing-related subsidies […] would make a big difference to reducing risk of homelessness; and we want to be upstream about this, as opposed to downstream […]. Definitely, let’s get the bed bugs out, but let’s just keep building so that we have more subsidized housing in order to reduce that waitlist.

Resources that support the full spectrum of housing needs, upstream (prevention) and downstream (Housing First) are crucial. For example, a participant from the women-specific workshop suggested that comprehensive support for single mothers should include both housing and childcare subsidies:

Low-income housing and childcare subsidy need to come together to be able to help single mothers out in these types of situations. Somehow, we need to figure out how we can intervene on people—on women and families—before it gets to that desperation…we need to catch it earlier.

Similarly, for youth, funding cuts were emphasized as a key determinant for long waitlists and limited resources and transitional supports and preventative interventions when aging out of the foster care system, as described by a participant from the youth-specific workshop:

The Ministry [of Child and Family Development] could provide funding for [youth awareness] programs because that’s often where the problem begins. If you have the support early on, the issues can be mitigated before they actually become issues.

One way in which participants suggested combatting that stigma and discrimination was to hire people, including peers, who live and work in the community. Doing so would enable providers to better relate to youth, seniors, cisgender and transgender women, diverse ethnocultural groups (e.g., Indigenous, Chinese, Indian), people living with mental health challenges, and people who are low-income. Moreover, this would enable clients to build stronger rapport, feel more supported, and achieve self-sufficiency according to a participant from the women-specific workshop.

We have LGBTQ+ [lesbian, gay, bisexual, trans, queer and others] groups and a couple of centres. I’ve actually just taken on a client who just transitioned [gender transitioning] and having a hard time with their family and the only thing I could do is hire a staff member who has transitioned, at a volunteer capacity going to meet up with him. But apart from that, there’s not a lot.

In addition, participants from the women-specific workshop suggested that immigrants with English language difficulties could be better supported by resourcing cultural brokers who have the communication capability and broad understandings of different cultural norms, values, beliefs, behaviors, and practices:

The language barrier is a big one. I’d try to find someone within our organization who speaks her language. Even if she speaks English, sometimes it’s better for people to speak their first language because they can express themselves so much easier in their own language and see what she needs first.

Developing cross-sector partnerships across housing, health, and social services was highlighted as way to create safer, securer living environments. For example, participants suggested having more integrated working and communication between non-profit organizations that support PEH and private rental agencies, landlords, and family members. Working with housing providers was described by a participant from the youth-specific workshop:

There’s a network called the Friendly Landlord Network, so there are some resources out there that are particularly renting out to youth. It really depends on what you get, but at least these landlords are actually housing youth, and they know what they’re signing up for once signing off the network, so it’s kind of a good thing right now.

Finally, participants described the need for self-awareness and empathy among those who have not experienced homelessness by “putting yourself in their situation,” as stated by a participant from which workshop from the youth-specific workshop. Service provider participants wanted to get a better understanding of the everyday realities of being homeless, to reduce social distance, and to work not only for PEH but with. A participant from the youth-specific workshop suggested this could be achieved by “getting into the community […], making those connections […], going to their place and getting a feel for what they do, how they live, and how we can work together.”

## Culturally-responsive planning resource to support housing first

Guided by cultural safety and humility principles, we developed a Culturally-Responsive Planning (CRP) resource, consisting of a model (Fig. 1) and tool (Table 1), to support the implementation of Housing First programs. The CRP model illustrates six principles – each underpinned by two recommendations for health and social care providers, public health practitioners, and planning professionals. The CRP tool provides direction for application in practice. The suggested execution of the resource is to first review Housing First principles, and to implement these with reference to CRP principles (Fig. 1). Second, key cultural safety and humility considerations, reflexive questions for responsive-planning, and suggested resources can be applied alongside each Housing First and CRP principle (Table 1). Of note, we were careful to not reinvent existing resources, but scoped and reviewed existing tools across international contexts and reference these resources within the tool.

## Discussion

This study examines: “How can tenants of cultural safety and cultural humility be applied to support sub-populations of youth, older adults, and women who are experiencing homelessness?“ To answer this question, we used cultural safety and humility lenses to identify ways to support youth, older adults, and women experiencing homelessness who require distinct considerations for housing and support to enhance the Housing First approach.

Subsequently, our analysis has shed light on some of the limitations of the Housing First approach for distinct sub-populations underlining the necessity for more responsive and tailored strategies to address the heterogeneous needs of individuals experiencing homelessness. Our interpretation of the findings contributes to the broader discussion on Housing First’s effectiveness for various sub-populations in several following ways.

According to recent evidence, the evolving landscape of homelessness has seen a shift in the demographics of those affected, with certain sub-populations, such as youth, older adults and women, becoming increasingly likely to experience homelessness [3–7]. This change in the PEH is influenced by various factors, including the aging of the general population, socioeconomic disparities, and the scarcity of affordable housing [7]. For example, older adults face unique challenges when experiencing homelessness, such as increased vulnerability to health issues, difficulty navigating the housing and social services systems, and ageism [10]. Furthermore, older adults may have complex needs that are not addressed adequately by traditional homeless services, including specialized medical care, age-appropriate housing options, and assistance with daily living activities [10]. Similarly, youth experiencing homelessness also encounter unique challenges, such as disrupted education, lack of life skills and job experience, and increased vulnerability to exploitation and abuse [45, 46]. They require tailored services that focus on family reunification (when appropriate), skill development, education support, and trauma-informed care [45, 46].

Likewise, the At Home/Chez Soi Demonstration Project has previously called for the need to modify the Housing First approach for different sub-populations, including Indigenous communities, racialized communities, and rural communities [17]. Our findings support this calling and contributes to recent literature by examining service providers’ perspectives on the cultural appropriateness and responsiveness of the Housing First approach for supporting several sub-populations of PEH in Metro Vancouver and tailoring these to population specific needs.

Accordingly, heightened rates of sub-populations facing unique challenges substantiate the need to adapt Housing First approaches to serve distinct groups with unique needs more effectively. In response to participants’ experiences, such adaptations may involve:

1. Developing age-specific Housing First models that cater to the unique needs and challenges of older adults and youth, ensuring that they receive appropriate support and services that address their distinct circumstances.
2. Enhancing collaboration between housing, health, and social service providers to ensure a comprehensive and coordinated response to the needs of youth, older adults, and women experiencing homelessness.
3. Expanding the availability of age-appropriate, and gender-specific and affordable housing options, with a focus on creating supportive housing environments for women, women with children, and older adults and transitional housing options for youth.
4. Prioritizing preventative measures that address the root causes of homelessness for sub-populations, including eviction prevention, early intervention programs, domestic violence and family mediation services for at-risk youth.
5. Promoting ongoing research and evaluation of Housing First initiatives targeting sub-populations to identify best practices, challenges, and opportunities for improvement.

Participants’ insights from this study have helped to enrich the conversation around Housing First’s efficacy for distinct groups, such as older adults, and stress the importance of preventative measures to avert the negative repercussions of homelessness for these sub-populations, while accentuating the need for adapting the Housing First approach to cater to the specific needs of diverse sub-populations with intersecting identities.

It is clear from our analysis that to successfully house and support diverse sub-populations of PEH, deeper appreciation of barriers to services and the cultural (in)appropriateness and (non)responsiveness of services and systems is imperative. In view of this, the importance of cultural safety and cultural humility in the development and implementation of culturally-appropriate and -responsive health and social care programs, including Housing First initiatives has not surprisingly been emphasized in past literature [22, 26, 47, 48]. These concepts are essential in addressing the unique needs and experiences of diverse populations, such as youth, older adults, and women experiencing homelessness.

Cultural safety refers to an environment where individuals feel respected, valued, and safe from cultural harm or discrimination [27]. It involves recognizing and addressing power imbalances, prejudices, and systemic barriers that may impact marginalized populations [27]. In the context of Housing First initiatives, this means ensuring that service providers are aware of and sensitive to the cultural backgrounds and experiences of those they serve.

Cultural humility, on the other hand, is an ongoing process of self-reflection and learning that enables service providers to be open, respectful, and adaptable when working with diverse populations [31]. It acknowledges that no single cultural perspective is universally applicable and encourages service providers to engage in a continuous process of learning and adaptation to best serve the unique needs of each individual [31].

Findings of this study analyzed according to the concepts of cultural safety and cultural humility offers deeper understandings into how service providers perceive the implementation of Housing First initiatives for specific sub-populations, such as youth, older adults, and women experiencing homelessness. These insights reveal the challenges and opportunities that exist in tailoring Housing First programs to better serve diverse populations, underscoring the importance of integrating cultural safety and cultural humility into such initiatives.

According to our interpretation of the findings, in order to effectively incorporate cultural safety and cultural humility in Housing First initiatives, several steps can be taken:

1. Providing ongoing cultural competence training and education for service providers to enhance their understanding of and sensitivity to the unique needs of diverse populations.
2. Encouraging open dialogue and collaboration between service providers and community members to foster a deeper understanding of the specific cultural factors that may influence an individual’s experience with homelessness and their engagement with support services.
3. Developing and implementing policies and procedures that prioritize the principles of cultural safety and cultural humility, ensuring that all aspects of Housing First initiatives are designed and delivered in a culturally-responsive manner.
4. Actively engaging diverse populations in the planning, implementation, and evaluation of Housing First programs, ensuring that their voices and perspectives are heard and considered in the decision-making process.
5. Continuously evaluating and refining Housing First initiatives to ensure they remain responsive and adaptive to the changing needs of diverse populations experiencing homelessness.

Subsequently, informed by the analysis, a key output was a CRP resource consisting of a model and a tool. The model illustrates six principles for considering whether homelessness services are culturally-responsive. The model is accompanied by a tool consisting of recommendations, considerations, questions, and additional resources to support health and social care providers, public health practitioners, and planning professionals. Providing essential culturally-responsive care to diverse populations of PEH involves molding existing structures and systems to enable and empower wellness for distinct sub-cultures, and tailoring culturally-responsive planning solutions to promote positive housing and wellbeing outcomes when applying Housing First.

The CRP resource emphasizes the importance of cross-sectoral partnerships that work across housing, health, and social service sectors to create safer, more secure living environments and centralized and tailored resources for clients [10]. Notably, this may inspire a ‘total community effort’ akin to a multisystem approach seen in mental healthcare for youth [49], through the meshing of culturally-responsive housing and supports to empower youth motivation, enable older adults to age well in place, support women to feel safe and confident, and keep families together.

### Strengths, limitations, and future directions

A key strength of this study relates to how the findings have served to address one of the key limitation of Housing First, which requires more attention on some of the diverse needs of homeless subgroups (e.g., emancipated and other youth, women with or without children, older people [5, 50–54]). Our interpretation and analysis produced nuanced understandings of varying unmet needs among PEH through a cultural safety and humility lens to reconceptualize data acquired from a metropolitan centre that has high rates and diverse experiences of homelessness.

However, regarding limitations, first, this body of knowledge can be further enhanced. Future research should also examine the diverse needs of racial and ethnic sub-populations of PEH, including Indigenous and Black PEH who are overrepresented in homeless samples [3].

Second, in terms of population representation and insight, participants were mainly providers and participants needed to be over the age of 19, which is the legal age in the province of British Columbia, Canada. Consequently, some important lived experiences of PEH under age 19 may have been excluded, including those of harder to reach PEH. Such experiences may have offered deeper comprehension of unique everyday realities of homelessness to add richness to the analysis and further support the development of the CRP resource. The research team acknowledges that if culturally-responsive planning is to be a model approach, a future study which makes prominent the voices of clients to inform the further development of the CRP resource is required. This would also enable verification of the interpretation of the data and implications for the CRP resource.

Third, there is international variation (i.e., US, United Kingdom, Europe and Australia) in how Housing First policies and programs are operationalized in terms of their design and administration. In this study, the way in which Housing First was implemented focuses on housing related supports and services to address homelessness within the Canadian context. Future research can expand on this work by exploring the design and administration of the range of Housing First programs and policies and how operational factors of Housing First functions to influence housing, health, and wellbeing outcomes for diverse clients, across other geographical and cultural contexts.

Despite these limitations, a key and innovative contribution of this study to the field is the development of the CRP resource which can be used in community, health, and social service planning. Although the CRP resource was developed based on a study conducted in Metro Vancouver, it can be applied flexibly across Western urban settings experiencing similar challenges of homelessness, including the United States, Canada, Australia, the United Kingdom, and countries across Europe. We recommend that the CRP resource be used in close collaboration with the local community and across professional sectors. We also propose an evaluation of this resource across different environments with priority on the perspectives of individuals with lived experience; and envision forthcoming research to examine how this resource can be applied in resource-scarce locations, such as lower- and middle-income countries and in rural and remote regions.

To evaluate the CRP resource effectively and ensure its usefulness, relevance, and applicability across various contexts, we propose a combination of pre- and post-implementation surveys, focus groups and interviews and case studies. For pre- and post-implementation surveys, a questionnaire can be circulated among housing, health, and social service providers before and after implementing the resource to assess changes in knowledge, attitudes, and practices related to culturally-responsive planning. Alongside this, focus groups and interviews can be conducted to engage diverse stakeholders, including individuals with lived experience, service providers, and community leaders, in focus groups and interviews to gather qualitative feedback on the resource’s strengths, weaknesses, and areas for improvement. Last, the application of the resource in various settings (such as urban, rural, and remote regions, as well as lower- and middle-income countries) can be examined through case studies. Applying this method can enable identifying of context-specific challenges and strategies for adapting the resource to diverse environments.

By employing mixed evaluation methods, the effectiveness and relevance of the CRP resource can be better understood and improved upon, ultimately contributing to more tailored and effective support for diverse populations experiencing homelessness.

## Electronic supplementary material

Below is the link to the electronic supplementary material.

Supplementary Material 1

## Data Availability

Additional data can be found in supplementary files 2–4. Raw data can be supplied upon request by emailing the corresponding author MLF.

## Abbreviations

CBPR: Community-based participatory research
CRP: Culturally-responsive planning
GVSS: Greater Vancouver Shelter Strategy
LGBTQ+: lesbian, gay, bisexual, trans, queer and others
PEH: people experiencing homelessness
