# Supplementary material for: Supporting intersecting cultural needs of gender and age by increasing cultural safety and humility for Housing First initiatives
Source: BMC Public Health. 2023 May 30;23:1005. doi: 10.1186/s12889-023-15955-7 (PMC10227820; doi:10.1186/s12889-023-15955-7)
Supplement: Supplementary file 1 — Supplementary Material 1 [file 12889_2023_15955_MOESM1_ESM.docx]

Supplementary File 1: Coding Framework

| **Theme 1** | **Insufficient built environments create challenges across gender and age** |
| --- | --- |
| **Code** | **Description** |
| Transportation | Transportation challenges as it pertains to youth (e.g., safety issues travelling at night) from one location to another (e.g., one service location to another, travelling to shelter); older adults (e.g., living with chronic condition and / or mobility issues); and women (e.g., safety considerations at night, challenges travelling with a child). |
| Safety and security | Safety and security issues of youth, older adults, and women experiencing homelessness and living in shelters |
| Universal access | Youth, older adults, and women have equal opportunities to participate in any housing system and services (as it pertains to homelessness) |
| Age-specific built environment needs | Refers to the needs of youth or older adults as it pertains to the surrounding built conditions of temporary housing and/or homelessness shelters (e.g., to facilitate mobility needs) |
| Gender-specific built environment needs | Refers to the needs of women as it pertains to the surrounding built conditions of temporary housing and/or homelessness shelters (e.g., separate bathrooms, toilets, etc.) |
| Urban and rural resource considerations for youth, older adults, and women | Refers to the respective considerations for different needs of youth, older adults, and women residing in urban vs rural locations |
| Green space | Refers to an area of grass, trees, or other vegetation set apart for recreational or aesthetic purposes in an otherwise urban environment that surrounds temporary housing and shelters |
| Walkability | Refers to amenities within a 5–10-minute walk from temporary housing and shelters |
| Access to local amenities | Refers to accessible and available amenities necessary for youth, older adults, and women (e.g., GP office, pharmacy, grocery stores, childcare, cafés and coffee shops, convenience store) that surrounds temporary housing and shelters |
| Multi-faceted care environments | Refers to care environments that support different people living with different challenges with different needs (e.g., detox, trauma support, child welfare, counseling, dental care) and tailored to youth (e.g., support for mental health, education), older adults (e.g., dementia care), and women (e.g., support for access feminine care, birth control) |

| **Theme 2** | **Cultural safety and humility considerations at the intersection of gender, age, and housing insecurity** |
| --- | --- |
| **Code** | **Description** |
| Structural inequities & disadvantages | In relation to issues of ‘ housing/homelessness’: experiences of an embedded bias in organizations, institutions, governments or social networks (by homeless people) which creates advantages for some members and marginalizes or produces disadvantages for other members |
| Stigma and stigmatization | Negative perceptions, attitudes, prejudice against and stereotypes of homeless people specifically within the context of health care and social service provision |
| Power imbalance | Difference in power between service providers and users (typically when service providers have more decision-making power that determines the livelihood and wellbeing of those with less power) i.e., disempowerment of homeless people |
| Culture of youth, ageing, or being a woman | Ideas, customs, and social behavior of a particular group of people in the case of youth, older adults, or being a woman |
| Intersectional Inequality | Experiences of different and more than one layer of inequality to housing supports and services i.e., older adult woman or younger women or lone mother |
| Motherhood or Single motherhood | Homelessness and homelessness services and support access challenges associated with being a mother or a single mother |
| Gender roles & responsibilities | Generalized roles and responsibilities that are thought to be possessed by or assumed by women or men that impact homelessness and homelessness services and supports |
| Unmet support needs of older adults | Unmet needs of older homeless people i.e., in addition overarching needs of homeless people such as housing is the most essential need, followed by the need for regular meals and adequate clothing and physical needs that revolve around access to health care: medical, surgical, and dental services and substance use treatment. |
| Unmet support needs of youth | Unmet needs of younger homeless people i.e., in addition overarching needs of homeless people such as housing is the most essential need, followed by the need for regular meals and adequate clothing and physical needs that revolve around access to health care: medical, surgical, and dental services and substance use treatment. |
| Unmet support needs of women | Unmet needs of homeless women i.e., in addition overarching needs of homeless people such as housing is the most essential need, followed by the need for regular meals and adequate clothing and physical needs that revolve around access to health care: medical, surgical, and dental services and substance use treatment. |

| **Theme 3** | **Supporting culturally-responsive Housing First implementation** |
| --- | --- |
| **Code** | **Description** |
| Tailored resources and supports | Homelessness services and supports made or designed for a particular person such as youth, older adults, and women |
| Knowledge and skills transfer for housing service providers | Refers to any recommendations that can inform Housing First as it pertains to opportunities for cultural safety and humility education, information, explanations, teaching, and training for housing service providers |
| Partnership working | Refers to any recommendations that can inform Housing First as it pertains to instances of working together from two or more different organizations and/or services working jointly together to facilitate integrated service provision |
| Changes in working practices | Refers to any recommendations that can inform Housing First as it pertains to impact and outcome in terms of changes to working practice of homelessness service providers |
| Changes in structural processes | Impact and outcome in terms of changes to policies and organization of homelessness services |
| Equity in service provision | Distribution of homelessness services, equity in access to services, and service distribution to youth, older adults, and women |
| Culturally-safe and responsive care | Refers to any recommendations that can inform Housing First as it pertains to historical and political contexts that have facilitated structural violence and inequity; opportunities to reinforce and promote inclusivity, through focusing attention on structural inequities and disadvantages experienced by ethnic minority homeless youth, older adults, and women |
| Considerations for diversity of homelessness experiences | Refers to any recommendations that can inform Housing First as it pertains to considerations for the different experience and needs of youth, older adults, and women |
| Youth guidance and moral support | Refers to any recommendations that can inform Housing First that relate to having adult role models, guidance, and moral support for youth |
| Early childhood development | Refers to any recommendations that can inform Housing First as it pertains to needs of homeless youth in relation to early development stages: Early childhood, middle childhood, and adolescence represent the 3 stages of child development. |
| Safe living environments | Refers to any recommendations that can inform Housing First as it pertains to creating / promoting safer temporary housing accommodations for youth, older adults, or women |
| Person-centered approach | Biopsychosocial & holistic approaches, opportunities for health promotion, person choice |
| Enhanced Cultural knowledge | Having knowledge and understanding of Ideas, customs, and social behavior of a particular group of people in this case youth, older adults, and women to shape ‘Housing First’ |
| Safeguarding physical and mental harm | Primarily refers to: protecting children from abuse and maltreatment and preventing harm to children's health or development; however, in this case, also with consideration for safeguarding in relation to the situation of older adult and considerations for women |

Supplementary File 2: Concepts and data – Insufficient built environments create challenges across gender and age.

| **Thematic Concepts (TC)** | ^a^**Supporting Quotations (SQ)** |
| --- | --- |
| 1. Age-related built environment needs | 1. “Housing First being the priority for how the federal government is funding housing, one of the problems of how the housing-related is seniors would have to become homeless first. I’m not an expert […] but, as I understand it, as I was talking to staff doing this work, the Housing First doesn’t really benefit seniors. Persons would have to become homeless first to be able to benefit from this. That is, as I see it, is a real flaw in the system. You want to be better able to support a person who is on fixed income, who has income but still cannot afford to live where they are, and know what we can do to help them stay at home, so that they don't become somebody who is homeless and then have to access services; especially if you think about the cost of that vs. the cost of somebody who remains in their home.” |
| 1. Gender-specific built environment needs | 1. “There’s not enough women-only services and adding to that is using the broad category of “women” erases communities within that category [e.g., elderly women, immigrant and refugee women, Indigenous women, trans women]. Sometimes women-only services aren’t adequate enough […] you need something even more specific.” 2. “I’m finding more lately that we’re getting transgender people. It’s becoming more common and I’m finding it really hard to get our transgender women or men help because it’s either men or women. I’ve actually had certain places say, no we won’t help you because you’re not living 24 hours as a woman.” 3. “I think as long as there’s violence happening, you need to have women-specific resources. You can’t just tell them, ‘get along’, and the violence goes away.” |
| 1. Access to affordable housing | 1. “From what I can see, it’s about access to housing. So, there’s that overall lack of affordable housing that poses a barrier for any typical family to access.” |
| 1. Safety and security | 1. “Many of them said that they would rather live on streets and sleep on the street rather than going to a shelter […] because they think that some shelters are very dangerous. I have some single senior women who went to a shelter. She felt unsafe.” |
| 1. Transportation needs | 1. “The entire challenge around transportation makes it difficult for women to get out and about.” 2. “When you look at it – the Downtown East Side – all those resources; then you look at Greater Vancouver, there are not many options […]. People are going on a regular basis, every day, to those resources, rather than travel to many different places where they don't even have any transportation.” |
| 1. Urban and rural resource considerations | 1. “We need one-stop shop instead of spread-out resources. There is value in other communities taking pressure off one main community having it all, but it’s hard having it in your backyard. It’s okay to be clustered, but it has to make sense, like don’t have a recovery house next to a liquor store. Don’t have transition houses next to alleyways where violence happens. Temptation is everywhere, and yet you’re expected to recover and do so successfully.’ |

^a^Note: The list of supporting quotations is not exhaustive.

Supplementary File 3: Concepts and data – Cultural safety and humility concerns at intersection of gender, age and housing insecurity.

| **Thematic Concepts (TC)** | **^a^Supporting Quotation(s)** |
| --- | --- |
| 1. Housing and health related service and support needs | 1. “There are some services where people go into isolated senior's homes to make sure they have all their benefits, if they need a doctor [we help find one]. The difficulty is finding isolated seniors; it's very difficult. We're thinking of ideas—putting notices in doctor's offices and different places where seniors go. […] we have outreach workers who we call ambassadors, and they seek out seniors, to try to find isolated seniors." 2. "I did become homeless with my children […] I found it hard to even get into an emergency shelter with my children. Women who are fleeing abuse or become homeless leaving a relationship – [it] is really hard for them to even get into a shelter. There are waiting lists for shelters now." |
| 1. Cultural considerations for women | 1. “Sometimes women at a young age will turn to prostitution because when they age out [of the foster care system], or when they turn 18 with a family in foster care, if they’re in low-income housing, they have to pay rent or otherwise they have to get out. I was in a situation where I lived with my mom. She was on assistance my whole life. And then I turned 18, and they said I needed to pay, or I need to move somewhere else. And I actually called escort places thinking this is my only option. I was only 18 years old. And this is a common thing. Our young girls become targets at a young age.” 2. “I think culture is another big barrier. I know that in Indian culture, it’s not normal to leave […] the abusive relationships. Or, even if it’s not necessarily abusive, it’s not a good or healthy relationship; it’s still really hard to leave.” |
| 1. Cultural considerations of youth | 1. “From my experiences dealing with youth, it’s too much all at once and then they just shut down, so that’s why there might be some struggles for school. So, I make sure this youth is involved in their plan, because a lot of the times, up until 19, everything is made up for them—you’re gonna do this, you’re gonna do that—and then it’s kind of like the ball is dropped and they’re like, ‘I don’t know what to do here.’ So, I think for this case, I’d really make sure she wants to continue, make sure that’s something she wants to do. That’s the biggest thing, just hearing the youth, ‘What do you wanna do? I know you’ve been told what you should do, but what do you actually wanna do?’” 2. “We meet a lot of people who are 18 and are about to age out of the system, they've been ignored for years.” |
| 1. Cultural considerations for older adults | 1. “Some family members dropped this older woman off [at the hospital], because they said they couldn’t take care of her anymore. But, she had no real health issues besides maybe some dementia […]. We used to see a lot of elder abuse, financial especially, taking their money and then just dropping them off.” 2. “They [family members] dropped them [an older family member] off at the hospital, especially in the emergency room. I also worked in the ER. They said they couldn’t cope and didn't know how, so they would drop them off for us to take care of them. They would stay in the hospital until we can find a way for them to go back home. It’s not necessary for them to be in the hospital and they’re actually at-risk for catching other things […]. They’re also at-risk for falling.” |

| 1. *Gender roles & responsibilities* | 1. “Who knows where she can go? This [is] what I do when somebody comes to me, I bring them to A, B, C. Sometimes they just need that, ‘Okay, let’s meet up, and I will bring you here.’ Sometimes these women don’t even know the city at all. I’m able to bring them, and I have bus tickets for them, but there are not a lot of workers. There are other issues with their children, too. They could be at-risk of being taken. So, then they don’t want to reach out to these places [shelters] because what if their kids are taken because they’re homeless, because they’re putting their kids at risk? That happens a lot.” |
| --- | --- |
| 1. *Layers of intersectional, social inequality* | 1. “I don’t like saying it, but the reality is for First Nations people, they aren’t making it to 65 if they’ve been abusing substances.” 2. “I have one senior who is male, he comes out of a shelter every morning—I think before 10 o’clock—and he is wandering around. He doesn’t want to go back there because his stuff is being stolen by people in the shelter, so during the day, he had a disease, so he goes to the hospital regularly. […] he had a very challenging time. His stuff has been stolen and he was the only person who spoke Mandarin in that shelter, no one speaks other languages other than English.” |
| 1. *Power imbalance* | 1. “It’s frustrating dealing with hospitals. The nurses and the doctors need some kind of training on sensitivity […]. And, even sometimes the paramedics. There are some paramedics who are amazing […], but some paramedics who are really disrespectful to women, we’ve seen it. It’s terrible how the women get treated. I’ve accompanied the women to the hospital before and I’m just appalled […]. It’s so degrading.” 2. “I think within like mental health services or child and youth mental health services, obviously, there’s a wait time –there’s a whole referral process and sometimes you have to retell your story of trauma or whatever over and over to your counsellor or to your GP [general practitioner] or to your psychiatrist. I think you just lose faith in the system and trust.” |
| 1. *Discrimination and stigmatization* | 1. “What I’ve noticed is there’s a lot of stigma of youth who have been in [foster] care. When I call in, I’ll be honest, I don’t say it’s for youth. I’ll be, like ‘Oh, you got a suite available, can I come and see it?’ and then I’ll go with the youth. And we actually provide a letter, saying they’re in our program and they are engaged in our program, and we do check-ins, we do program planning, things like that. So, it has helped, but I still feel that there is a lot of pull-back from landlords.” 2. “I saw a news release before, and it said the number one thing landlords don’t want to rent to is single moms. The next one is First Nations people. The next one was Asian people. And I think the other one was people on income assistance. And I was like, no wonder I’m not getting any housing, I’m a single mom, I’m First Nations, I could pass for Asian, and I’m on income assistance. I had so much against me.” |
| 1. *Structural housing inequities & disadvantages* | 1. “A lot of people cannot afford. And they put down [demolish an] old building […] they renovate it, get people out evicted, say ‘Okay, you can come back. I’ll give you two months as a bonus. You leave a building and come back in the future after we renovate it.’ And after they come back, they actually have to pay extra 20% more and they cannot afford that.” 2. “People are being priced out of Vancouver, so we’re having to refer people outside of Vancouver for services.” |

^a^Note: The list of supporting quotations is not exhaustive.

Supplementary File 4: Concepts and data – Supporting culturally-responsive Housing First implementation.

| **Identified Concepts (TC)** | **^a^Supporting Quotation(s)** |
| --- | --- |
| 1. Changes to structural processes | 1. “Increased funding for housing-related subsidies […] that would make a big difference to reducing risk of homelessness; and we want to be upstream about this, as opposed to downstream. […] definitely let’s get the bed bugs out—but let’s just keep building so that we have more subsidized housing in order to reduce that waitlist. The waitlist is years and years long at present.” 2. “I think low-income housing and childcare subsidy need to come together to be able to help single mothers out in these types of situations. Somehow, we need to figure out how we can intervene on people, on women and families, before it gets to that desperation. How can we, as people in a community, stop this from happening before it happens? This is where I go back to — we need to catch it earlier. So, this woman has anxiety, why does she have anxiety? What can we do to help her with her anxiety so she can keep her job and doesn’t wind up in the system? I feel strongly about that.” 3. “They are very active with trying to connect with youth and give them what resources are available to them. But the problem is that they’re running out of funding. I used to do my other practicum there, and they had to start letting people go. I’m worried about talking to anybody there, because every time I talk to them, they lose their job.” |
| 1. Changes to frontline work practices | 1. “[What] comes to mind is having a youth worker involved who is a stable and consistent person so that they can notice the first time that they’re using drugs or when they’re starting to hang out with that person and the changes and really being aware and sensitive to that, and then plugging in and asking those questions so that it’s not just the youth doing her thing and finding that place to belong alone. They have someone walking with them.” 2. “I mean, she’s housed. It’s at 19, when everything falls apart, that’s when they show up at our door. I noticed that social workers are way overworked. I know they have a huge caseload, but I think it would be helpful if you start like a year in advance preparing this person for what’s about to change, what’s about to happen.” 3. “Twenty-four-seven accessible detox intake for women, and it’s immediately going to happen. No waitlist! 24/7 emergency mental health interaction, intervention, support. Decent, safe, respectful hospital-based care for women. In order for women to go to place, and feel safe and not judged” 24/7 women-only victim services – afraid to call the police because they don’t want the situation to get worse.” |
| 1. More culturally- responsive care | 1. “We have a lack of representation of Aboriginal staffing, so that’s something that we are looking at. Like at least we have like representation or something. It’s always good to have our staffing right so that they can connect.” 2. “Advocacy, education, and network, connecting seniors to groups from the similar cultural backgrounds and helping seniors who have language barriers and programs that encourage seniors to get out to.” 3. “We have LGBTQ groups and a couple of centers. I’ve actually just taken on a client who just transitioned [gender transitioning] and having a hard time with their family and the only thing I could do is hire a staff member who has transitioned, at a volunteer capacity going to meet up with him. But apart from that, there’s not a lot.” |
| 1. Enhanced partnership working | 1. “There’s a network called the Friendly Landlord Network, I don’t know if you’ve heard of it yet, so there are some resources out there that are particularly renting out to youth, so I try to. It really depends on what you get, but at least these landlords are actually housing youth, and they know what they’re signing up for once signing off the network, so it’s kind of a good thing right now.” 2. “It’s important for adult children [to be] recognizing their parents’ signs or symptoms of dementia, forgetfulness, the forgetfulness; is it dementia or is it not, is it normal age-related changes? Just creating or promoting awareness and understanding the disease so that they can better support their parents.” 3. “Only we can connect them to service providers, other services providers to get them to access to the services. Sometimes it’s really hard because we don’t see seniors coming out. So, we don’t know the problems. If the seniors attend our programs and suddenly disappear, [but] there are so many for us to call. We have no time; we receive service provider one-to-one calls already, so we cannot offer to call everyone who are absent from the programs. So, that’s why we need a lot of volunteers like senior peers or other volunteers to do that.” |
| 1. Promoting safe and secure living environments | 1. “Housing, specifically housing for women with children; perhaps private market rentals, but affordable and easily accessible. Housing with bigger units. I know BC Housing [local social housing association] have a supplemental program for women with children, but the waitlist is a joke. There waitlist is on the waitlist of waitlists […] it could be a private market rental, but still affordable and easily accessible with bigger units too, where are women with six children supposed to go? You can't fit them into a 3-bedroom apartment.” 2. “In our assisted independent living department, all of the homes need to ensure it’s safe for the client to live in—at least two exits, and they’re not locked, making sure that there’s a window in the bedroom; and the window you can open should a fire or any other emergency situation occur. That they can exit a room. Making sure that the home is safe; and the care provider’s role is to ensure safety of the individual and that includes the home.” |
| 1. Tailoring resources and supports | 1. “I would like to see low-barrier ‘wet’ type housing. I frequently have clients who are facing eviction because of their alcohol use problems. And if they’re supported in other ways, maybe they could perhaps keep drinking or may be more motivated to decrease their use of it if they had the proper supports in place. But, if you boot them out, they haven’t got any supports.” 2. “That is very typical to get someone who has high anxiety. Male or female, but we’re focusing on women. A lot of the people that we deal have high anxiety and they can’t hold down a job because of whatever mental health issues are there. And it may or may not be related to addiction, but it’s something that you have to drill down and find out — what is that barrier? And, to figure out how to help them get through that barrier so they can find meaningful employment.” 3. “The language barrier is a big one. I’d try to find someone within our organization who speaks her language. Even if she speaks English, sometimes it’s better for people to speak their first language because they can express themselves so much easier in their own language and see what she needs first.” 4. “Youth awareness program(s). Ministry [of child and family development] could provide funding for these programs because that’s often where the problem begins. If you have the support early on, the issues can be mitigated before they actually become issues.” |

^a^Note: The list of supporting quotations is not exhaustive.
